# Immobilized Horseradish Peroxidase on Discs of Polyvinyl Alcohol-Glutaraldehyde Coated with Polyaniline

**DOI:** 10.1100/2012/129706

**Published:** 2012-04-24

**Authors:** Samantha Salomão Caramori, Kátia Flávia Fernandes, Luiz Bezerra de Carvalho Junior

**Affiliations:** ^1^Unidade Universitária de Ciências Exatas e Tecnológicas, Universidade Estadual de Goiás, Caixa Postal 459, Rodovia BR 153, Km 9875132-903 Anápolis, GO, Brazil; ^2^Departamento de Bioquímica and Laboratório de Imunopatologia Keizo Asami (LIKA), Universidade Federal de Pernambuco, Avenida. Prof. Morais Rego, Campus Universitário, 50670-910 Recife, PE, Brazil; ^3^Laboratório de Química de Proteínase, Departamento de Bioquímica e Biologia Molecular, Instituto de Ciências Biológicas, Universidade Federal de Goiás, Caixa. Postal 131, 74001-970 Goiânia, GO, Brazil

## Abstract

Discs of network polyvinyl alcohol-glutaraldehyde (PVAG) were synthesized and coated with polyaniline (PANI) using glutaraldehyde as a chemical arm (PVAG-PANIG-HRP disc). The best conditions for the immobilization were established as about 1.0 mg mL^−1^ of protein, for 60 min and pH 5.5. The soluble enzyme lost all of its activity after incubation at 70°C for 15 min, whereas the PVAG-PANIG-HRP disc retained about half of the initial activity for pyrogallol. The same PVAG-PANIG-HRP disc was used consecutively three times without any activity lossbut presented 25% of the initial activity after the 7th use. PVAG-PANIG-HRP disc retained approximately 80% and 60% of its initial activity after 60 and 80 days of storage, respectively. Resorcinol, m-cresol, catechol, pyrogallol, **α**-naphthol, **β**naphthol, and 4, 4′-diaminodiphenyl benzidine were efficiently oxidized by the PVAG-PANIG-HRP disc (from about 70% to 90%), and it was less efficient towards aniline, phenol, and 2-nitrosonaphthol.

## 1. Introduction

Peroxidases (Enzyme Commission number 1.11.1.7) are a huge family of heme-containing enzymes that catalyze oxidation and reduction reactions of large families of substrates.

Their broad substrate specificity, polyfunctionality, and availability from different sources allow their application in various biotechnological processes. Oxidative destruction of colored compounds is significantly stimulated by peroxidase and it is a practical interest for decoloration processes used in food, textile, and paper industry as well as in washing powders [1, 2]. Peroxidases have a variety of biotechnology application due to this catalytic mechanism. For instance, they are capable of oxidizing many compounds bearing the functional group R-N=N-R′ (azo-dyes), in which R and R′′ can be either aryl or alkyl groups. These compounds are widely used in the textile industry and discarded in water collections. They are defined as pollutants because of their mutagenic and carcinogenic effects. Additionally, azo-dyes can cause biological cycles alteration [3, 4]. Peroxidases are also extensively used in clinical and immunological analysis, such as glucose [5, 6], cholesterol [7, 8], and urea [9] determinations, procedures involving H_2_O_2_ releasing. Horseradish peroxidase (HRP) is the most popular source among the peroxidases. It can use a variety of organic compounds as donors and acceptors of electron.

The immobilization of these enzymes has been used to enhance the potential applications of them. Immobilized peroxidase obtained from bitter gourd (*Momordica charantia*) has been reported to present more advantages than the free enzyme in the treatment of wastewater contaminated with phenols [10]. Immobilized *Saccharum spontaneum* peroxidase [11] and potato polyphenol oxidase [12] have also been proposed to decompose textile dyes.

 Previously in our laboratories, HRP was successfully immobilized on powder of PANIG [13, 14]. This enzymatic derivative presented difficulties to be separated from the reaction mixture. Attempts to overcome the separation step were also conducted by our research group, culminating in the preparation of PET-PANIG composite as strips with immobilized HRP, which could be manually removed from reaction medium. However, this material presented low protein loading [15].

PVAG has been used for protein immobilization because of the plasticity and high surface area of this material [16–18]. The hydrophilic behavior of PVA, on the other hand, interferes in the microenvironment of the immobilized protein when nonpolar substrates/products are involved. Moreover, comparisons of PVA and PVA-PANI composites indicated a less immobilization capacity of PVA when it is not covered by PANI [19]. Trying to overcome the limitation of PANI powder and PET-PANI composites, and using the interesting properties of PVA, discs of PVAG covered with PANI are proposed as a matrix for HRP immobilization. The resulting composite was investigated to propose an alternative immobilized HRP preparation to be used in the detoxification of wastewater containing phenolic compounds. Some features affecting the immobilization procedure and properties of this derivative were then investigated.

## 2. Materials and Methods

### 2.1. Materials

Pyrogallol and catechol were obtained from Sigma-Aldrich (Saint Louis CA, USA). Alpha- and beta-naphthol, m-cresol, resorcinol, and phenol were purchased from Vetec Química Fina Ltda. (São Paulo SP, Brazil). Aniline, 2-nitrosonaphthol, 4,4′ diamine-diphenyl-benzidine were purchased from Merck (Darmstadt, Germany). HRP was kindly provided by Dr. Elba Bon (Universidade Federal do Rio de Janeiro, Rio de Janeiro, Brazil).

### 2.2. PVAG-PANIG Discs Synthesis

The PVAG-PANIG disc synthesis was based on procedures described by Carvalho et al. [17] and Fernandes et al. [13], modified by Caramori et al. [19]. Briefly 2% (w/v) polyvinyl alcohol (10 mL) was dissolved at 65°C, and then 2.5% (v/v) glutaraldehyde (1.5 mL) was added in order to form a PVA-glutaraldehyde network [19]. Aliquots (20 *μ*L) of this mixture were transferred to 96 wells of a microplate containing 3.0 M HCl (120 *μ*L) and kept at 25°C for 24 h, after which the discs were formed (PVAG synthesis). Afterwards, the discs were removed and incubated with 0.61 M ammonium persulphate, prepared in 2.0 M HCl, for 30 min. Then, they were incubated with 0.44 M aniline for 60 min (PVAG coating with polyaniline: PVAG-PANI). Finally, the PVAG-PANI discs were activated using 2.5% (v/v) glutaraldehyde for 60 min and exhaustively washed with 0.1 M phosphate buffer, pH 6.0 (PVAG-PANIG synthesis). This process yielded 240 discs (wet) weighting about 15 mg each.

### 2.3. HRP Immobilization

One disc of PVAG-PANIG was incubated with HRP (1.0 mL containing 12,500 U mL^−1^; 7 *μ*g mL^−1^ prepared in different solutions of 0.1 M sodium acetate buffer at pH 4.0–5.5 or phosphate buffer at pH 6.0–7.5) for 60 min, under orbital shaking (80 rpm) at 4°C. Afterwards, the immobilized enzyme on the HRP-PVAG disc, from now on abbreviated as PVAG-PANIG-HRP, was washed 3-folds with 0.1 M sodium acetate or phosphate buffer, twice with 1.0 M NaCl and twice with the same buffer. Discs of PVAG were used to immobilize HRP in the same conditions described above. HRP activity was not detected in the final washing buffer.

### 2.4. Enzyme Determinations

The activities of the soluble and immobilized HRP were established according to Halpin et al. [20] using pyrogallol and H_2_O_2_ as substrates. Briefly, either one disc of PVAG-PANIG-HRP (15.0 mg) or 0.1 mL of soluble HRP (874 U mL^−1^) was incubated at room temperature (28°C) with 0.5 mL of 12.69 mM pyrogallol (0.5 mL), 5.0 mM H_2_O_2_ (0.25 mL) prepared in 0.1 M sodium acetate buffer, pH 4.5, or 0.1 M sodium phosphate buffer, pH 7.0 (1.4 mL), and after one min (soluble HRP) or 10 min (PVAG-PANIG-HRP) the formed product (purpurogallin) was spectrophotometrically established at 420 nm (Ultrospec 2000, GE Health Care). One unit of enzyme activity (*U*) was defined as the amount of enzyme capable of oxidizing one micromole of pyrogallol per minute under experimental conditions (*ε* = 961 M cm^−1^).

### 2.5. Immobilization Optimization Conditions

The PVAG-PANIG-HRP disc was synthesized as above described using enzyme concentration varying from 0.502 to 0.912 *μ*g mL^−1^. Increasing amounts of HRP prepared in 0.1 M sodium phosphate buffer, pH 7.0 were incubated with the PVAG-PANIG disc for 60 min at 4°C. The derivative was washed 3-folds with 0.1 M sodium acetate buffer pH 5.0, followed by twice with 1.0 M NaCl and twice with the same buffer. The PVAG-PANIG-HRP disc activity was established and the retained protein estimated by the difference between offered protein and that found in the washing solutions. Firstly, the optimum pH was established incubating samples of soluble HRP with PVAG-PANIG discs, in the appropriated buffer (4.0–5.5 using 0.1 M sodium acetate buffer and 6.0–7.5 using 0.1 M sodium phosphate buffer). After that, the incubation time (30–210 min) was optimized. After washing the PVAG-PANIG-HRP discs their activities were measured as described above. All of these experiments were performed in triplicates and the results expressed as mean ± standard deviation.

### 2.6. Immobilized HRP Properties

The following properties of the soluble and immobilized HRP (preparation synthesized under the best conditions) were investigated: the time course of the pyrogallol oxidation, the activity of the preparations as above described at pH varying from 3.5 to 7.0 (3.5–5.5 using 0.1 M acetate buffer and 6.0–7.0 using 0.1 M sodium phosphate buffer) and at temperatures varying from 30°C to 60°C. Soluble HRP (0.1 mL) or one disc of PVAG-PANIG-HRP were incubated with 12.69 mM pyrogallol (0.5 mL), 5 mM H_2_O_2_ (0.25 mL) either at the indicated pH, 28°C, or at the indicated temperatures, pH 4.5. Then, after either 1 min (soluble) or 10 min (immobilized), aliquots were withdrawn and the formed product (purpurogallin) spectrophotometrically established at 420 nm. One enzyme unit (U) was defined as the amount of HRP capable of forming 1 *μ*mol of purpurogallin per min.

 The thermal stability of both free (0.1 mL) and immobilized enzymes (one disc) was established by incubating the preparations at 50°C and 70°C. Samples were then withdrawn at time intervals (15–45 min) and after equilibration at 37°C their activities were determined as described above. Kinetics was evaluated by assaying the preparation activities at pyrogallol concentrations varying from 4.0 to 48.7 mM. The HRP activity versus pyrogallol concentration was plotted. In order to estimate apparent Vmax and Km, a nonlinear regression (hyperbolic fit) was performed, using Sigmaplot 2000, version 6 (Systat Software Inc., USA). The reuse of the immobilized enzyme was evaluated by assaying the same preparation seven times and washing it with buffer (0.1 M sodium acetate, pH 4.5) between each procedure. Finally, the shelf life was analyzed by keeping several PVAG-PANIG-HRP discs in 0.1 M acetate buffer, pH 4.5, at 4°C and assaying the stored disc activity every seven days up to 80 days. The 100% value was established as the activity at the initial incubation time. All of these experiments were performed in triplicates and the results expressed as mean ± standard deviation. Figures 2–6 were plotted using Sigmaplot 2000, version 6 (Systat Software Inc., USA).

### 2.7. Treatment of Phenolic Compounds by the Soluble and PVAG-PANIG-HRP Disc

The soluble enzyme (0.1 mL) and PVAG-PANIG-HRP (one disc) containing both similar activity (about 90 U) were incubated at 37°C for 1.5 h with 0.1 mM sodium acetate buffer (2.0 mL), pH 4.5, containing 0.75 mmol H_2_O_2_ and 1.0 mM of the following phenolic compounds: resorcinol; m-cresol; catechol; pyrogallol; aniline; phenol; *α*-naphthol; *β*-naphthol; 4,4′-diaminodiphenyl benzidine; 2-nitrosonaphthol. Afterwards, the remaining phenolic compounds were spectrophotometrically (750 nm) established according to Lowry et al. [21] and compared with the amount found in the untreated phenolic solutions (100%). All these experiments were performed in triplicates and the results expressed as mean ± standard deviation. 

## 3. Results and Discussion

**Figure 1:**
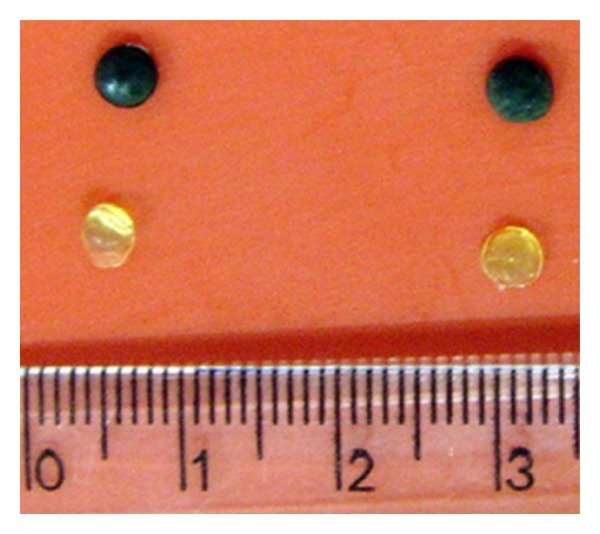
Disc of polyvinyl alcohol-glutaraldehyde-PVAG (yellow) and coated with polyaniline-PVAG-PANIG (dark-green).

The PVAG-PANIG disc synthesis is carried out in two steps: firstly, a network of polyvinyl alcohol molecules is formed using glutaraldehyde as an arm under acid catalysis [16, 19] yielding the disc, which is coated with polyaniline chemically synthesized from aniline [13]. The PVAG and PVAG-PANIG discs presented 4.0 mm in diameter due to the microplate wells used as mold (Figure 1).

Thus the HRP is covalently fixed onto the PVAG-PANIG disc via glutaraldehyde. It is important to notice that the physical shape of the PVAG occurs depending on the template used during the synthesis. Here, the wells of a microplate were used and therefore discs were obtained. However, PVAG was already used for plasticizing filter paper, which was used as a matrix for protein immobilization [18]. The moldability, flexibility, biocompatibility, and adhesive property of PVA make it a versatile material for bioengineering. Recovering PVA with PANI increases the superficial area of this composite material and hence the protein loading, as mentioned by Caramori et al. [22].

**Figure 2:**
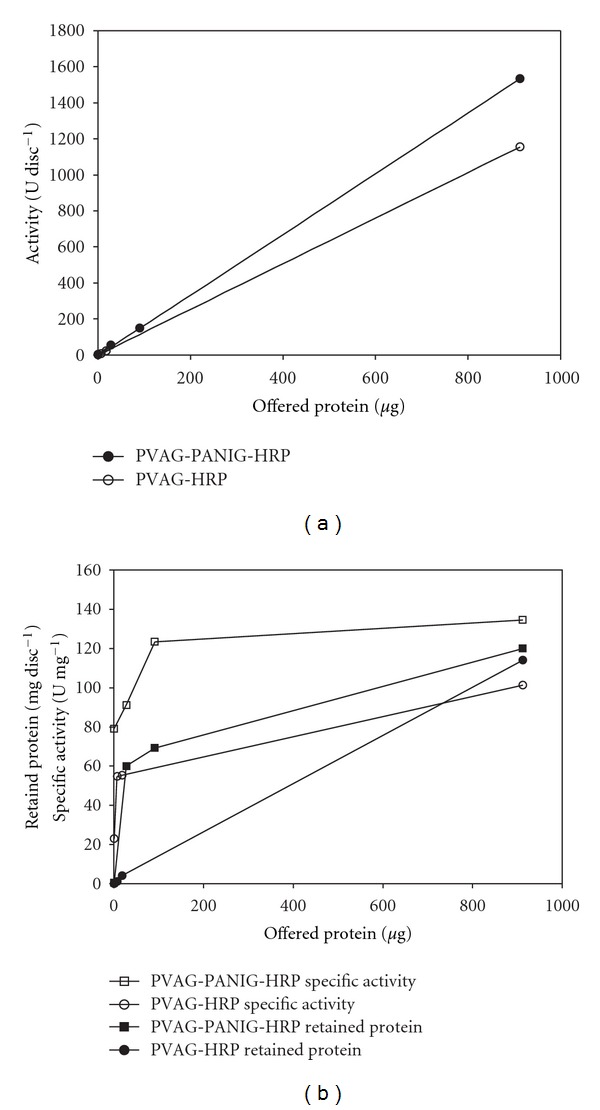
Relationship between offered and retained activity (a), protein (b),and specific activity (b) of the immobilized HRP on the PVAG-PANIG disc.

Figure 2 shows the relationship between the offered HRP and the immobilized enzyme expressed in terms of retained activity, protein per disc, and activity per mg of retained protein (specific activity). It is worthwhile to notice that the PVAG disc itself has carbonyl groups available [16, 17] for the HRP immobilization. However, the glutaraldehyde-treated PANI coat would increase these available groups as demonstrated by the increased activity of the PVAG-PANIG-HRP disc compared to that found for the PVAG-HRP preparation (Figure 2(a)). This is also reflected by the difference on the specific activity between them (Figure 2(b)). Intriguingly, the difference in terms of retained protein is negligible (Figure 2(b)). Therefore, one can admit that the PANI coating provides some advantage to the PVAG disc as far as the HRP immobilization is concerned. According to this result there, is a linear relationship between the offered HRP and the catalytically active immobilized HRP per disc (Figure 2(a)). However, this correlation is not followed by the specific activity (Figure 2(b)), which showed a hyperbolic curve suggesting that there is a limit after which inactive enzyme molecules are immobilized. Steric hindrance due to the overloading could be probably attributed to this effect. To avoid this phenomenon, all experiments to characterize the immobilized enzyme were carried out using a preparation synthesized with 7.0 *μ*g mL^−1^ of the soluble HRP instead 1.0 mg mL^−1^.

Recovering of PVAG with PANIG improved its capability for HRP immobilization 24.6% (PVAG 1,155 U disc^−1^ against PVAG-PANIG 1,533 U disc^−1^). The effectiveness of this material can be better demonstrated when comparing it with PET-PANIG, another composite tested for HRP immobilization [15]. PVAG-PANIG presented 9,650 more active immobilized HRP than PET-PANIG (PVAG-PANIG = 1,219,935.540 U cm^−2^ versus PET-PANIG = 126.41 U cm^−2^). Caramori et al. [22], based on UV-Vis spectra, surface area porosimetry, and scanning electron microscopy analyses, reported the PVAG-PANIG disc as a macroporous structure. The authors concluded this macroporous aspect due to PANI coating.

**Figure 3:**
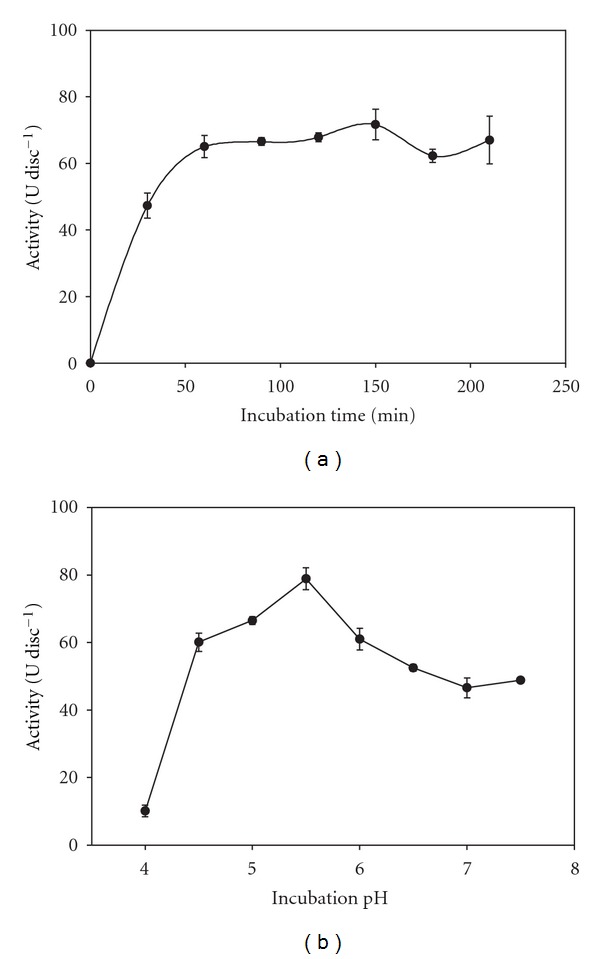
Optima time (a) and pH (b) for the immobilization of the soluble HRP on the PVAG-PANIG.

Figure 3(a) shows that 60 min is the best incubation time for the derivative PVAG-PANIG-HRP synthesis, after which longer incubation does not increase the catalytic activity of the immobilized enzyme. This time depends on the support and/or chemical groups involved in the HRP immobilization on different materials [23, 24]. In the case of PANIG, the immobilization of HRP requires generally 60 min [6, 13].

The best pH immobilization value of 5.5 for the HRP immobilization on PVAG-PANIG is shown in Figure 3(b). This pH value also depends on the support and/or chemical groups involved. Fernandes et al. [13] immobilizing HRP on PANIG reported a different optimum immobilization pH (6.0). This discrepancy can be probably attributed to the PVAG influence.

The time course of the pyrogallol oxidation catalyzed by the soluble and PVAG-PANIG-HRP disc showed that under the established experimental conditions a semilog plot (first order kinetics) was attained at the 1st and 10th minute, respectively (data not shown). Furthermore, all substrate was oxidized after 30 min of incubation by using both preparations.

**Figure 4:**
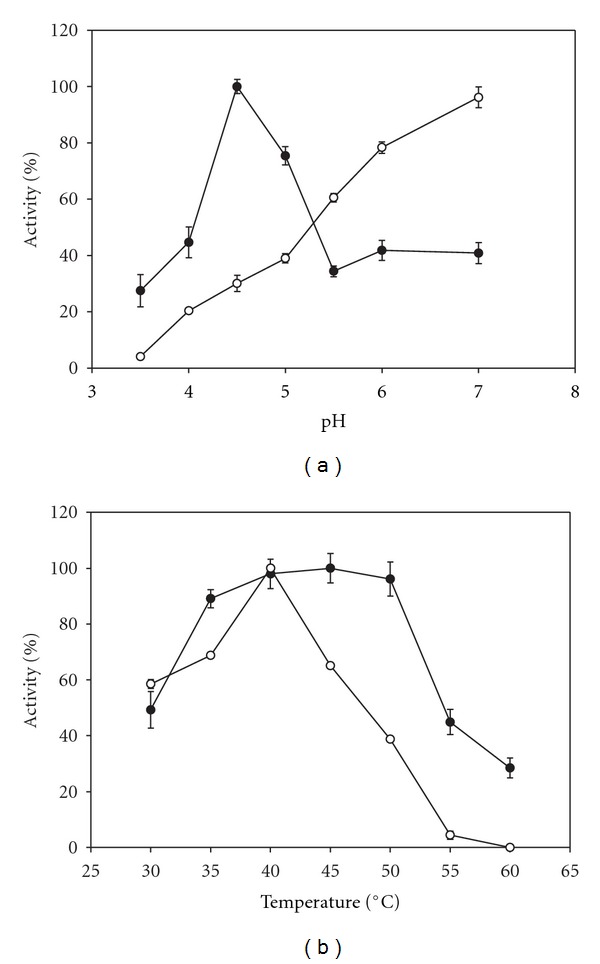
Influence of the pH (a) and temperature (b) on the soluble (∘) and PVAG-PANIG-HRP (●).

The pH profiles for the soluble and PVAG-PANIG-HRP activities are displayed in Figure 4(a). Higher pH values than 7.0 were not studied because of the pyrogallol “autoxidation”. There is a marked difference of the pH influence on the enzyme activities. The immobilized enzyme presents optimum pH value at 4.5 whereas the soluble form at approximately 7.0 as already reported in the literature [25]. Fernandes et al. [14] reported a value of 7.0 for the HRP immobilized on PANI. Negatively and positively charged matrices are known to displace the pH profile towards to alkaline and acid pH values, respectively, as compared to the native enzymes, at low ionic strength. This left shift of optimum pH for the PVAG-PANIG-HRP suggests that the PVAG-PANIG presents positive charges on its surface. Li and Townshend [26] also reported different optimum pH for the HRP activity before (7.0) and after immobilization (5.8) on polytetrafluorethylene tubing.

**Figure 5:**
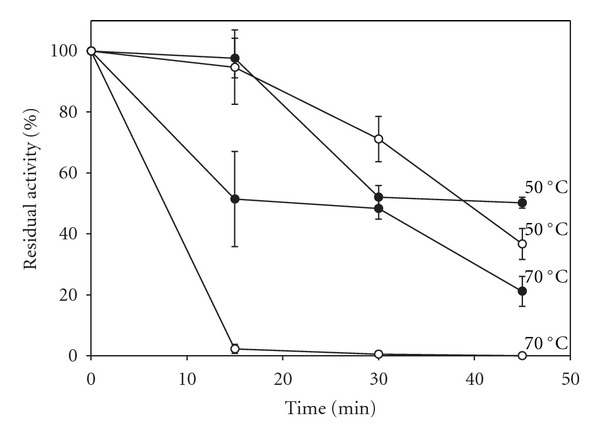
Thermal stability at 50°C and 70°C for the soluble (∘) and PVAG-PANIG-HRP (●).

The effect of the temperature on the soluble and PVAG-PANIG-HRP is shown in Figure 4(b) and optimal temperatures were found to be 40°C and 40–50°C, respectively. The decays of the descending arms of the curves denote different effects of the temperature on the soluble and immobilized enzyme. No activity was detected for the soluble enzyme assay at 60°C, whereas the immobilized enzyme assay still presented some activity (30% of that at 50°C). Similar results were reported by El-Essi et al. [27] and Lai and Lin [28] for HRP immobilized on sol-gel matrices and porous glass, respectively. Further evidences for the higher thermal stability for the PVAG-PANIG-HRP can be seen in Figure 5 which showed that the soluble enzyme activity decreased faster than the immobilized preparation when both were incubated at 50°C and 70°C. The soluble enzyme lost all activity after 15 min at 70°C, whereas the immobilized one retained about 20% of its initial activity. Increase of the thermal resistance after HRP immobilization has already been reported in the literature [24, 29–31].

The action of the soluble and PVAG-PANIG-HRP on pyrogallol followed the Michaelis-Menten kinetics. Values of apparent Michaelis constants were estimated to be 5.47 ± 0.71 mM and 12.07 ± 1.35 mM for the soluble and immobilized HRP (apparent value), respectively. This discrepancy showed to be statistically different (*t* = 1.92 and *P* = 0.03). Conformational/steric, partitioning, diffusional/mass-transfer, and microenvironmental effects are well known to influence immobilized enzyme kinetics. Kinetic parameters, particularly, the apparent Km, can provide the degree of these interferences when compared to that calculated for the soluble enzyme. This apparent constant usually increases as the result of impairment of the enzyme action under those effects.

**Figure 6:**
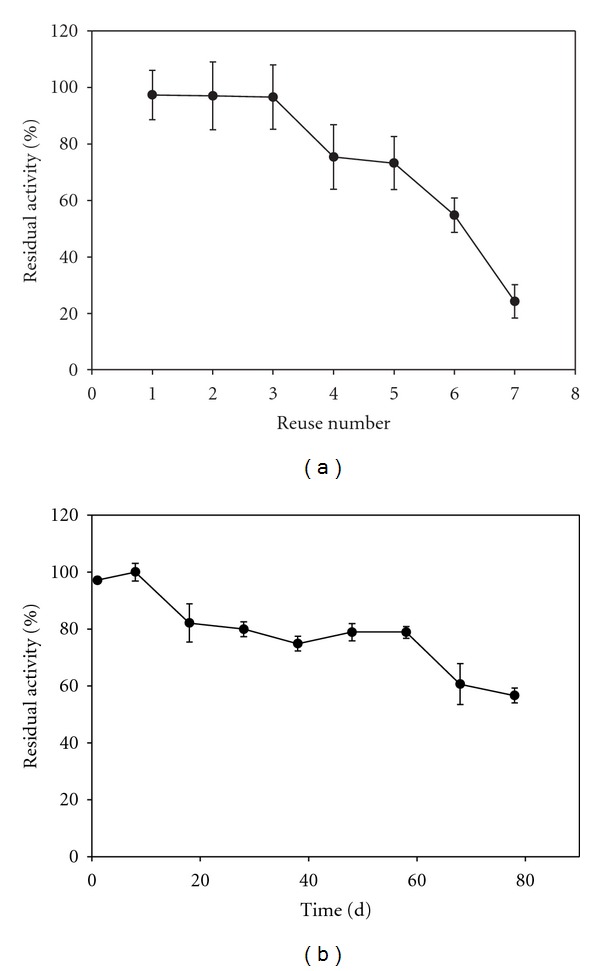
Reuse (a) and shelf life (b) of PVAG-PANIG-HRP.

The reuse of the PVAG-PANIG-HRP is presented in Figure 6(a). The same disc was used three times without activity loss and retained 25% of the initial activity after the 7th use. It is worthwhile to draw attention to the fact that this preparation was incubated with H_2_O_2_, a traditional HRP inhibitor and protein denaturing agent. The HRP derivatives (aluminum-pillared interlayered clay-HRP with addition of polyethylene glycol) synthesized by Cheng et al. [32], showed to be not reusable were and Akhtar et al. [33] reported poorer performance reusing immobilized bitter gourd peroxidase on Sephadex G-50. The storage stability (shelf life) of the PVAG-PANIG-HRP was investigated for 80 days at 4°C in 0.1 M sodium acetate buffer, pH 4.5 and Figure 6(b) resumes the results. The immobilized enzyme derivative retained approximately 80% and 60% of its initial activity after 60 and 80 days of storage, respectively. This behavior is superior to those reported by Rojas-Melgarejo et al. [34] and Cheng et al. [32].

The enzymatic approach has attracted much interest in the decolorization/degradation of textile and other industrially important dyes from wastewater as an alternative strategy to conventional chemical, physical and biological treatments [35]. Cheng et al. [32] removed, after 4 h, more than 90% of phenol using polyethylene glycol as aluminum-pillared-interlayered clay-peroxidase stabilizer in the reaction medium. The ability of the soluble HRP and PVAG-PANIG-HRP disc to remove phenolic compounds up to 1.5 h is demonstrated in the Table 1. Most of them were efficiently oxidized by both soluble and immobilized enzyme and no stabilizer was added. Pyrogallol, *α*-naphthol, catechol, *β*-naphthol, 4,4′-diaminodiphenyl benzidine, m-cresol, resorcinol, and 2-nitrosonaphthol were oxidized by the PVAG-PANIG-HRP disc in percents varying from 70% to 90%, except for 2-nitrosonaphthol (c.a. 18%). As mentioned above, pyrogallol was completely oxidized after 30 min of incubation by both enzymatic preparations. Therefore, the reusability and higher thermal stability of the PVAG-PANIG-HRP disc would offer advantage compared to the soluble enzyme. The PVAG-PANIG-HRP disc showed to be more efficient than the immobilized bitter gourd (*Momordica charantia*) peroxidase on Con A adsorbed-Sephadex G 50 [10]. According to these authors, resorcinol, m-cresol, catechol, pyrogallol, under similar experimental conditions, were only removed 18%, 11%, 45%, and 0%, respectively. 

Both enzymatic preparations showed to be less efficient towards aniline and phenol. However, the immobilized HRP presented better performance than soluble form. Different results were obtained with the immobilized bitter gourd peroxidase, which was more efficient to remove phenol (about 90%) than PVAG-PANIG-HRP disc (about 35%).

## 4. Conclusions

More than 200 discs of the network polyvinyl alcohol-glutaraldehyde (PVAG) coated with polyaniline (PANIG) can be synthesized by a simple and inexpensive procedure. HRP can also be covalently fixed onto these discs via glutaraldehyde (PVAG-PANIG-HRP disc). The immobilization efficiency of this material was more than 9,000 fold higher than the obtained with PET-PANIG composite. The optimum pH of the PVAG-PANIG-HRP disc is lower than that reported for the soluble enzyme, whereas the optimum temperature is higher. The PVAG-PANIG-HRP disc is more thermal resistant than the soluble enzyme. The same PVAG-PANIG-HRP disc can be reused and stored for months without significant activity loss. Resorcinol, m-cresol, catechol, pyrogallol, *α*-naphthol, *β*-naphthol, and 4,4′-diaminodiphenyl benzidine are efficiently oxidized by the PVAG-PANIG-HRP disc (approximately about 60% to 90%), while aniline, phenol, and 2-nitrosonaphthol are less efficiently oxidized. The flexibility of PVA and the advantages obtained in the immobilization procedure make the PVAG-PANIG composite a possible target for applications in the treatment of wastewater.

## Figures and Tables

**Table 1:** Phenolic compounds oxidation by the soluble and HRP-PVG-PANIG.

Phenolic compounds	Oxidation (%)	
	Soluble HRP	HRP-PVAG-PANIG
Pyrogallol	92.1 ± 0.3	92.1 ± 1.1
*α*-Naphthol	87.6 ± 0.1	90.0 ± 1.2
Catechol	81.3 ± 0.4	86.5 ± 0.2
*β*-Naphthol	78.0 ± 0.2	83.2 ± 0.8
4,4′-Diaminodiphenyl benzidine	72.4 ± 1.8	75.1 ± 0.5
m-Cresol	62.0 ± 0.9	71.0 ± 0.4
Resorcinol	75.0 ± 0.3	70.8 ± 0.1
Aniline	28.4 ± 1.0	45.0 ± 3.7
Phenol	15.5 ± 0.9	35.8 ± 1.3
2-Nitrosonaphthol	18.8 ± 2.5	19.7 ± 2.4
